# Metagenomic sequencing reveals altered metabolic pathways in the oral microbiota of sailors during a long sea voyage

**DOI:** 10.1038/srep09131

**Published:** 2015-03-16

**Authors:** Weiwei Zheng, Ze Zhang, Cuihua Liu, Yuanyuan Qiao, Dianrong Zhou, Jia Qu, Huaijie An, Ming Xiong, Zhiming Zhu, Xiaohang Zhao

**Affiliations:** 1 Center of Basic Medical Sciences, Navy General Hospital, Beijing, China; 2 Third School of Clinical Medicine, Southern Medical University, Guangzhou, China; 3 School of Life Science, Tsinghua University, Beijing, China; 4 State Key Laboratory of Molecular Oncology, Cancer Institute and Hospital, Chinese Academy of Medical Sciences and Peking Union Medical College, Beijing, China

## Abstract

Seafaring is a difficult occupation, and sailors face higher health risks than individuals on land. Commensal microbiota participates in the host immune system and metabolism, reflecting the host's health condition. However, the interaction mechanisms between the microbiota and the host's health condition remain unclear. This study reports the influence of long sea voyages on human health by utilising a metagenomic analysis of variation in the microbiota of the buccal mucosa. Paired samples collected before and after a sea-voyage were analysed. After more than 120 days of ocean sailing, the oral microbial diversity of sailors was reduced by approximately 5 fold, and the levels of several pathogens (e.g., *Streptococcus pneumonia*) increased. Moreover, 69.46% of the identified microbial sequences were unclassified microbiota. Notably, several metabolic pathways were dramatically decreased, including folate biosynthesis, carbohydrate, lipid and amino acid pathways. Clinical examination of the hosts confirmed the identified metabolic changes, as demonstrated by decreased serum levels of haemoglobin and folic acid, a decreased neutrophil-to-lymphocyte ratio, and increased levels of triglycerides, cholesterol and homocysteine, which are consistent with the observed microbial variation. Our study suggests that oral mucosal bacteria may reflect host health conditions and could provide approaches for improving the health of sailors.

Seafaring has a long history, but sailors, who work under challenging conditions during long sea-voyages, have increased disease mortality and morbidity compared with land-based workers^1^. Historically, malnutrition, especially vitamin deficiency, was the main cause of disease on ships. The ocean milieu includes high humidity (90% to 100% on average), high salinity (35% on average), intense UV radiation, stormy waves, a monotonous environment, altered circadian biorhythms, sleep deprivation, and an insufficient supply of fresh fruits and vegetables, causing significant health-threatening physiological and psychological stress^2^,^3^,^4^. Although much attention has been paid to the health management of sailors during long sea-voyages, many areas of research remain unexplored.

Many commensal microbes live on different sites of the human body, participating in the host metabolic system, maintaining host immune system homeostasis and resisting pathogen colonisation^5^,^6^. The commensal microbiota encodes 200 billion unique coding genes, approximately 100-fold more than our own genome, and is thus sometimes known as the “second human genome”^7^. The US National Institutes of Health Human Microbiome Project (HMP) (http://www.hmpdacc.org) has characterised the microbiome in five major body sites from 242 healthy individuals^8^,^9^. By studying the variations within the microbiota, we are able to explore the impact derived from daily behaviours and environmental factors on these commensal communities and extend these findings to the interactions between the host and the microbiota^10^,^11^.

Classical microbial research techniques have been limited to culturable microbes, which only comprise approximately 10% of the total microbiota. In recent years, using high-throughput next generation sequencing (NGS) and bioinformatics analysis, metagenomic technology has greatly promoted the study of the human microbiome by enabling the detection of unculturable, low abundant and unclassified microorganisms^12^.

Mucosal surfaces are the primary environmental barrier for animals; thus, they are the main entry targets for pathogenic microorganisms. The oral mucosa is considered not only a barrier to the outside world but also an immunological and biochemical organ; therefore, oral health can be seen as a window to the body's health^13^. The nonspecific and specific immune activity of the mucosa constantly monitors and responds to oral cavity commensal organisms to maintain oral homeostasis and defend against potential pathogens. The Human Oral Microbiome Database (HOMD) (http://www.homd.org) was specifically designed to provide comprehensive information on oral bacteria and tools for examining and analysing each taxon in the human oral microbiome at both the taxonomic and genomic levels^14^. Such data can be used to speculate on the host physical condition by analysing the community structure and metabolism of the oral commensal microbes. Recently, it was suggested that the oral microbiome will cause disease in a majority of individuals during their lifetime and that aberrant microbial compositions are correlated with oral diseases, such as chronic periodontitis and saprodontia^10^,^15^.

In this study, we investigated the oral mucosa commensal microbes of sailors who engaged in an ocean voyage for more than three continuous months and studied the potential functional association between the microbes and host health using a new aspect of metagenomics. By combining DNA-based 16S rRNA phylotyping to identify the major bacterial phyla with whole genome shotgun sequencing (WGS) to obtain a deeper taxonomic analysis, we characterised the microbial diversity associated with host health and pathogens during a long-term ocean voyage. We found that after a long sea voyage, microbial diversity was reduced greatly, whereas several pathogens, such as *Streptococcus pneumonia*, increased. Furthermore, more than half of the microbial sequences were unclassifiable. Functional analysis revealed that sequences annotated as pertaining to microbial nutrient metabolism, such as folate biosynthesis and carbohydrate, lipid, amino acid and xenobiotic metabolism decreased significantly. Additionally, the belly-button microbiota were analysed and showed similar changes, including reduced microbial diversity and metabolic activities. The clinical laboratory examination of the host demonstrated decreases in haemoglobin and folic acid (FA) concentrations and the ratio of neutrophils to lymphocytes (GRN/LYM), and increases in the triglyceride (TG) and cholesterol (CH) levels, in accordance with the microbial variation. Our data are the first to suggest that the bacteria living in the oral mucosa are correlated with the host's health condition during sea voyages.

## Results

### Sequence data of the buccal mucosa commensal microbiota metagenome

To sequence the metagenome of the buccal mucosa commensal microbiota, we first performed 16S rRNA sequencing to illustrate the composition of the microbial community. On average, 55740 reads per buccal mucosa sample were obtained using the Illumina HiSeq2500, including 54219 that passed the quality control filter, with an average length of 253 nt (Supplementary Table S1). Only sequences that occurred at least five times were included in further analysis to preclude the inclusion of sequencing artefacts or potential contaminants from sample processing. As some degree of sequence divergence is typically allowed, a similarity of 97% is most commonly used in practice. By clustering the reads into operational taxonomic unit (OTU) at a 3% genetic difference, an average of 181 OTUs was obtained. In 16S rRNA sequencing, the taxonomic annotation revealed that half of the OTUs (50.00%) were identified at the species level and that 20.65% were identified at the genus level (Supplementary Figure S1 a).

**Figure 1:**
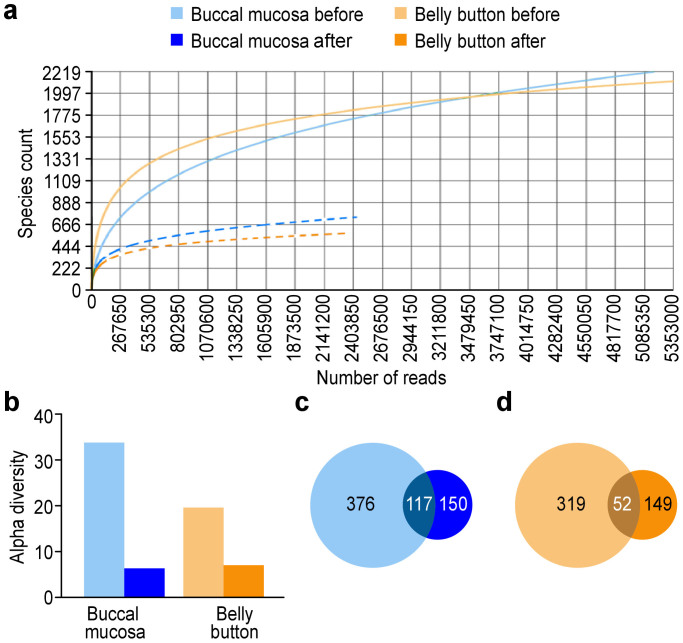
Microbial diversity is reduced after a long sea voyage. (a) Rarefaction curve of deep-sequencing samples for WGS. Dotted lines indicate the samples after the sea voyage. The rarefaction curve is derived from the protein taxonomic annotations. (b) Alpha diversity of deep-sequencing samples from the buccal mucosa and the belly button. The species richness was computed as the antilog of the Shannon diversity. (a and b) The data were analysed with the MG-RAST server using the Best Hit method. The data were compared with M5NR using a maximum e-value of 1e-5, a minimum identity of 80%, and a minimum alignment length of 15 (amino acids in protein databases and bp in RNA databases). (c) Venn diagram depictions of the buccal mucosa samples analysed by WGS. The number of taxa specific to the sample before (light blue) or after (dark blue) the sea voyage, and the number of taxa shared by both samples (deep blue) are shown. (d) Venn diagram depictions of belly button samples analysed by WGS. The number of taxa specific to the sample before (light yellow) or after (dark yellow) the sea voyage, and the number of classified species shared by both samples (deep yellow) are shown. (c and d) Taxonomic annotation was analysed with the MG-RAST server using the Lowest Common Ancestor method with the same cutoff values mentioned above. Colour code: light blue, buccal mucosa, before; dark blue, buccal mucosa, after; light yellow, belly button, before; dark yellow, belly button, after.

Because 16S rRNA sequencing distinguishes microbes at the genus level, we further employed WGS to obtain more details about the commensal microbial community. Samples from the study individuals were subjected to WGS. The sequencing resulted in a total of 3,804,224 sequences, with 794,145 sequences passing the quality filter discussed above. The average sequence length was 134 bp. Rarefaction curve analysis showed that the slope became flatter to the right, indicating that most of the species had been sampled (Figure 1a). To clarify the phylogenetic origin of the sequences, we determined the lowest common ancestor (LCA) by requiring that at least 80% of the genes had a best hit to the same phylogenetic group. This analysis showed that 16.63% of sequences had an LCA at the species level, 59.96% of sequences at the genus level, and 4.44% at the family/order/class/phylum level (Supplementary Figure S1 b). Unexpectedly, 18.97% of sequences had an LCA only at the domain level. Further analysis was performed later.

### WGS revealed that buccal mucosa microbial diversity decreased after a long sea voyage

First, we analysed the alpha diversity identified via WGS. The alpha diversity in the samples after a long sea voyage was significantly reduced (Figure 1 a and b) by approximately five times after a long sea voyage (33.85 to 6.26). By annotating the sequences using the NCBI taxonomy tree, we investigated the number of identified taxa in the two samples. In the before sample (before voyage), a total of 493 taxa were detected, whereas the number of detected taxa in the after sample (after voyage) decreased significantly to 267. Of the 643 total taxa detected in both samples, only 117 taxa were shared. There were 376 taxa specific to the before sample and 150 specific to the after sample (Figure 1c).

We also confirmed the change in the biodiversity at another body site before and after the sea voyage. Skin is the most exposed organ and, similar to the buccal mucosa, provides an open microenvironment for a large amount of microbes to live on. The belly button represents, to some extent, the natural state of the human skin microbial community because its unique position allows the resident bacteria to avoid daily removal by washing. Sequencing the belly button samples from the same volunteers who provided the buccal mucosa samples revealed that the microbial diversity in the belly button decreased more than two-fold after the sea voyage (19.91 to 7.08, Figure 1b). Accordingly, the number of taxa identified also decreased. There were 371 taxa in the before sample and 201 taxa in the after sample, with only 52 taxa being shared, indicating a great decline and change in the belly button microbial community (Figure 1d).

### 16S rRNA and WGS analysis of taxonomic variation in the buccal mucosa microbiota after a long sea voyage

**Figure 2:**
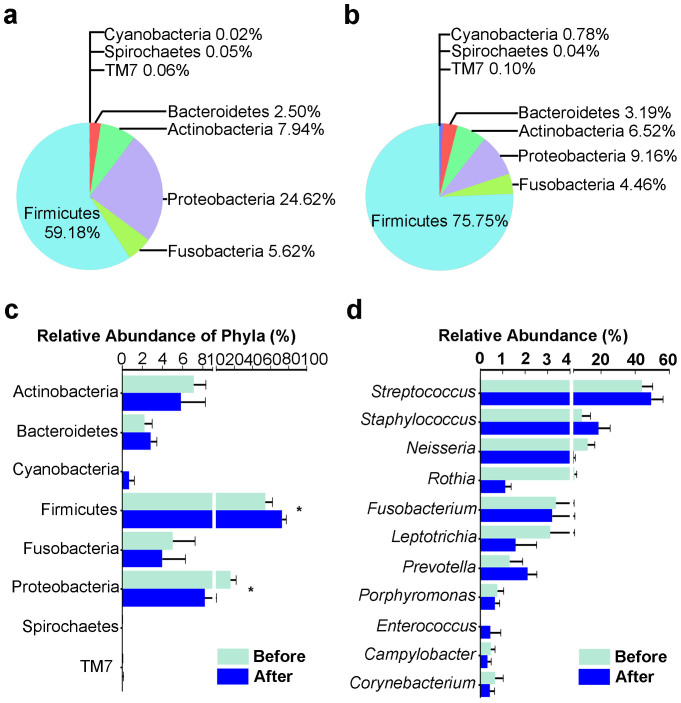
16S sequencing analysis of the variation in the buccal mucosa microbiome before and after a long sea voyage. (a and b) Pie chart of the relative abundance of the bacterial taxonomic hits at the phylum level in buccal mucosa samples before (a) and after (b) the voyage. The data are shown as the mean values. (c) Bar chart of the relative abundance of the bacterial taxonomic hits at the phylum level in the buccal mucosa before and after the voyage (*, *p* < 0.05. For Firmicutes, *p* = 0.0425; for Proteobacteria, *p* = 0.0122). (d) Analysis of the changes in the most abundant genera. Pairwise samples were collected at the time of setting sail and docking; n = 12. The data were analysed with the Qiime software package and the UPARSE pipeline. (c and d) The data are shown as the mean ± SD, Wilcoxon matched pairs test.

Next, we analysed the structure of the buccal mucosa microbial community. Phylogenetic analysis of the OTUs by 16S rRNA sequencing revealed that Firmicutes accounted for approximately half of the microbial community, consistent with a previous report^16^, and this was further increased after the sea voyage (Figure 2 a and b). After the sea voyage, the second richest phylum, Proteobacteria, decreased greatly, and the change in the abundance of Firmicutes and Proteobacteria was significant (Figure 2c). Then, the changes in the frequency of the most abundant genera were further analysed. *Streptococcus* and *Staphylococcus* were the most abundant genera, and their proportions increased after the sea voyage (*Streptococcus*, 44.23% to 49.61%; *Staphylococcus*, 9.05% to 18.71%). However, none of the changes reached significant (Figure 2d).

**Figure 3:**
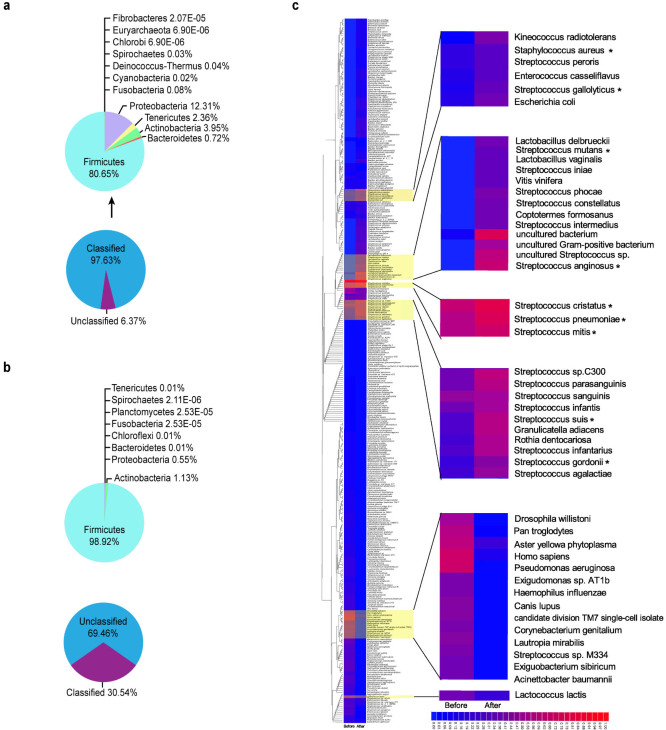
Variation in the taxonomic hit distribution after a long sea voyage analysed by WGS (buccal mucosa samples). (a and b) Pie chart of the percentage of unclassified sequences and the relative abundance of the MG-RAST taxonomic hits at the phylum level in buccal mucosa samples before (a) and after (b) a long sea voyage. (c) Heatmap depiction of the relative abundance of bacterial species from buccal mucosa samples. Colours reflect the relative abundance from low (blue) to high (red). The data were analysed using the Lowest Common Ancestor method with a maximum e-value of 1e-5, a minimum identity of 80%, and a minimum alignment length of 15 bp in RNA databases. Asterisks indicate species that are opportunistic pathogens.

WGS provides more information. At the domain level, sequences annotated as eukaryotes accounted for a quarter (26.05%) of the reads, and a small number of virus sequences (0.03%) were also detected. Within bacteria, Firmicutes was the most abundant phylum (80.65%), followed by Proteobacteria (12.31%) and Actinobacteria (3.95%) (Figure 3a), which is consistent with the results obtained by 16S rRNA sequencing. In the after sea voyage sample, bacteria composed up to 99.97% of the total reads. Surprisingly, unclassified bacteria accounted for 69.46% of the total bacteria (Figure 3b). The great number of unclassified bacterial sequences contributed largely to the sequences having an LCA only at the domain level mentioned above. Among those bacteria with known sequences, Firmicutes was the predominant bacterial phylum, accounting for 98.92% of the identified taxa. All other phyla decreased after the sea voyage: Actinobacteria accounted for 1.13%, and Proteobacteria for 0.55%. The percentages of the other phyla were no greater than 0.01%. Notably, the number of phyla identified decreased from 12 to 9.

At the genus level, *Streptococcus* was the most abundant genus, corroborating the 16S rRNA sequencing results. *Streptococcus* comprised 79.70% of the total identified bacteria in the before sea voyage sample and increased to 96.76% after the sea voyage. When analysing the taxa overlap between the two samples, the taxa identified after the sea voyage belonged mostly to *Streptococcus*, and fewer taxa belonged to *Clostridium, Listeria, Paenibacillus, Lactobacillus, Weissella, Enterococcus* and other genera. This finding was consistent with the result that *Streptococcus* was the most abundant genus in the buccal mucosa after the sea voyage. Notably, opportunistic pathogens such as *Streptococcus pneumoniae*, *Streptococcus anginosus*, *Streptococcus parasanguinis* and *Streptococcus suis* increased after the sea voyage, indicating a weaker host immune system (Figure 3c; Table 1).

### Comparative analysis of microbial KEGG pathways from samples before and after a long sea voyage

**Figure 4:**
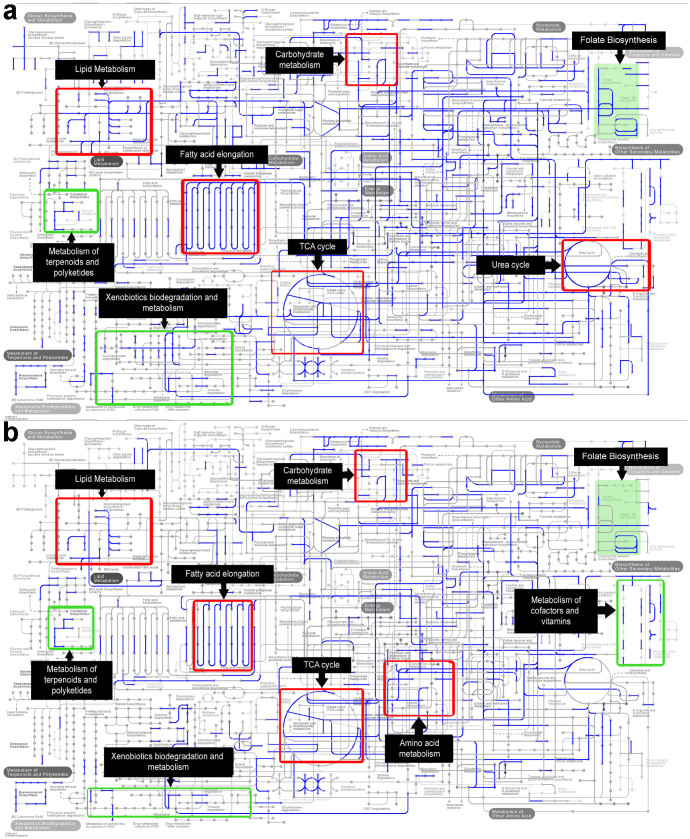
Projection of the metagenome onto KEGG pathways. Blue lines indicate pathways whose activity had decreased after the sea voyage. Representative pathways, particularly the folate biosynthesis pathway, are highlighted in green boxes. Red boxes indicate pathways involved in the metabolism of carbohydrates, lipids, or amino acids; green boxes indicate pathways that belong to other metabolic processes. (a) Buccal mucosa samples. (b) Belly button samples. The data were analysed with the MG-RAST server using the KEGG mapper for functional analysis with a maximum e-value of 1e-5, a minimum identity of 80%, and a minimum alignment length of 15 amino acids in protein databases.

The extensive non-redundant catalogue of bacterial genes provides opportunities to identify bacterial functions important for life under a long sea voyage in the marine environment. To clarify microbial functions, we annotated the function of enzymes identified in the whole-metagenome data according to the KEGG Orthology. The comparative analysis of microbial metabolic profiles between buccal mucosa samples before and after the long sea voyage showed a significant decrease in functional genes after the sea voyage, including three major metabolic pathways involving carbohydrates, lipids, and amino acids (Figure 4a). More specifically, in carbohydrate metabolism, pentose and gluconate interconversions, ascorbate metabolism, and upstream metabolism for the biosynthesis of the streptomycin, butirosin and neomycin were affected. In lipid metabolism, glycosphingolipid biosynthesis, arachidonic acid and ether lipid metabolism, and fatty acid elongation were affected. In amino acid metabolism, the urea cycle and cyanoamino acid metabolism were affected. Notably, the TCA cycle, which combines the three metabolic pathways above, was also reduced after the sea voyage. In addition to these basic essential metabolic pathways, other metabolic pathways were affected, including the metabolism of cofactors and vitamins (e.g., folate biosynthesis, biotin, thiamine, riboflavin and vitamin B_6_ metabolism), xenobiotic biodegradation and metabolism (e.g., benzoate, toluene, naphthalene, dioxin and aminobenzoate degradation and the metabolism of xenobiotics by cytochrome P450), and metabolism of terpenoids and polyketides (e.g., brassinosteroid and carotenoid biosynthesis).

The belly button microbiota showed a similar decrease in the genes involved in the metabolism of carbohydrates (e.g., ascorbate metabolism and upstream metabolism for the biosynthesis of streptomycin, butirosin and neomycin), lipids (e.g., glycosphingolipid biosynthesis, arachidonic acid and ether lipid metabolism, and fatty acid elongation) and amino acids (e.g., cyanoamino acid metabolism and the synthesis and biodegradation of lysine and ketone bodies). Moreover, genes involved in the metabolism of cofactors, vitamins (e.g., porphyrin, chlorophyll, and thiamine metabolism), terpenoids (e.g., brassinosteroid and carotenoid biosynthesis) and xenobiotics (e.g., metabolism of xenobiotics by cytochrome P450 and drug metabolism) were also decreased, which is similar to the results for the buccal mucosa samples (Figure 4b).

### Taxa profile in the belly button microbiota

Taxonomic analysis of the 16S rRNA sequencing suggested that the most abundant phylum in the belly button was Firmicutes followed by Proteobacteria, and the abundance of Firmicutes increased after sea voyage. At the genus level, *Staphylococcus* and *Corynebacterium* were the two most abundant genera, and the percentage of *Staphylococcus* increased, while *Corynebacterium* decreased after the sea voyage (Supplementary Figure S2). The WGS results showed that Firmicutes predominated in the belly button samples at both times (Supplementary Figure S3) and that the predominant genus shifted from *Streptococcus* to *Staphylococcus*. In addition, *Staphylococcus epidermidis* was the most abundant species after the sea voyage.

### The decrease in microbial folate synthesis positively correlates with serum folic acid concentrations of sailors

**Figure 5:**
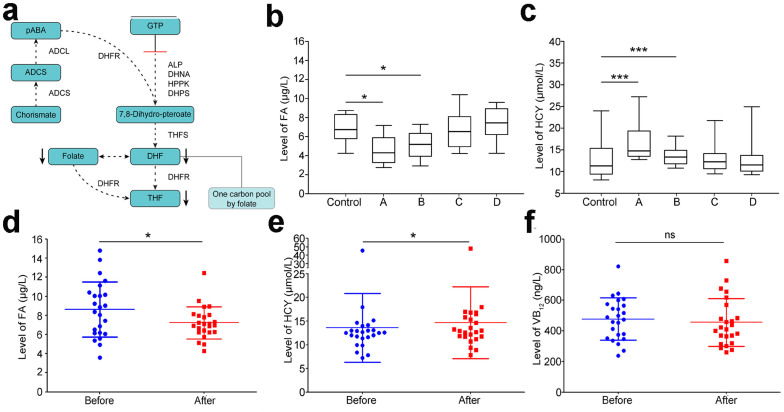
Folate biosynthesis decreases in both commensal bacteria and host serum. (a) Illustration of microbial folate synthesis in the buccal mucosa after a long sea voyage. Metabolites, boxed in black; enzymes, black; dotted arrows represent blocked pathways. DHF, 7,8-dihydrofolate; THF, 5,6,7,8-tetrahydrofolate; ADCS, 4-amino-4-deoxychorismate; PABA, p-aminobenzoic acid; ALP, alkaline phosphatase; DHNA, dihydroneopterin aldolase; HPPK, 2-amino-4-hydroxy-6-hydroxymethyldihydropteridine diphosphokinase; DHPS, dihydropteroate synthase; THFS, tetrahydrofolate synthase; DHFR, dihydrofolate reductase; ADCS, aminodeoxychorismate synthase; ADCL, aminodeoxychorismate lyase. (b and c) Comparison of serum folic acid (FA, (b)) and homocysteine (HCY, (c)) concentrations collected from different groups in Cohort 2, including a control group of onshore workers (n = 12): Group A, seafarers experiencing voyage without docking, n = 30; Group B, seafarers experiencing voyage without docking, n = 35; Group C, sailors experiencing voyage with docking, n = 50; Group D, sailors experiencing voyage with docking, n = 20. The p values are described in Table 2. *, p < 0.05; ***, p < 0.001, Mann-Whitney U-test. (d, e and f) Comparison of serum folic acid (FA, (d)), homocysteine (HCY, (e)) and vitamin B12 (VB12, (f)) concentrations of sailors from Cohort 1-Group 2 before (blue) and after (red) sea voyage. Pairwise samples were collected from sailors at the time of setting sail and at docking (n = 24). (d) p = 0.0471. (e) p = 0.0198. (f) ns, not significant; p = 0.1264, Wilcoxon matched pairs test.

Because the microbial metabolic analysis above showed that the folate biosynthesis activity decreased after a long sea voyage, we further analysed the change of bacterial folate biosynthesis in detail. Genes annotated as enzymes in the folate biosynthesis pathway were detected in the sample before the long sea voyage; detection of these enzyme in the after sample decreased significantly (Figure 5a). Two key enzymes in particular, dihydrofolate reductase (DHFR) and dihydropteroate synthase (DHPS), decreased more than other enzymes (DHFR, 45-fold reduction; DHPS, 74-fold reduction) (Supplementary Table S2). A decrease in detected folate biosynthesis enzymes indicated less microbially derived folate after the sea voyage.

To investigate the relationship between microbial folate synthesis, vitamin intake and the host serum level folic acid (FA), we investigated the serum FA concentration of sailors after different types of long sea voyages. Compared with a person who lives near a seashore (Cohort 2-Control Group), the serum concentration of FA decreased significantly during a long sea voyage with no seashore stops (Figure 5b; Table 2; Groups A and B; *p* < 0.01), and the decrease in FA improved when the sailors took vitamin complex tablets and ate plenty of fresh fruits and vegetables during the sea voyage (Cohort 2-Groups C and D). These findings indicate that insufficient vitamin intake is responsible for the decreased FA concentration in sailors and that a positive correlation exists between microbial folate biosynthesis and host FA levels.

Further investigation focused on the serum concentration of homocysteine (HCY) in the sailors. The concentration of HCY was higher in Groups A and B, showing a negative correlation between the FA and HCY concentrations (Figure 5c; Table 2). In Groups C and D, the increase in the HCY concentration was moderate as the level of FA increased. Research on vitamin B_12_ (VB_12_) did not reveal significant changes among the different groups.

Additionally, we further confirmed the variation in serum FA, HCY and VB_12_ levels of sailors experiencing the same voyage (from Cohort 1-Group 2, described in Methods). Pairwise samples were collected at the day of setting apart and docking. Consistently, after the sea voyage, the serum concentrations of FA decreased (from 8.64 ± 2.89 to 7.24 ± 1.69 (μg/L), Figure 5d), HCY increased (from 13.59 ± 7.24 to 14.69 ± 7.59 (μmol/L), Figure 5e), and no significant change was detected in the serum VB_12_ (from 477.7 ± 137.7 to 455.3 ± 156.7 (ng/L), Figure 5f).

### Evaluation of the physical condition of sailors using clinical laboratory examinations

To better study the relationship between the commensal microbiota and human health, we investigated several biochemical indices of the seafarer cohort. Fifty-seven individuals who experienced the same voyage as the volunteers underwent medical examinations before and after the long sea voyage. Their mean corpuscular haemoglobin concentration (MCHC) decreased from 332.57 g/L to 314.81 g/L, and their GRN/LYM decreased from 1.71 to 1.35; both of these changes were significant (Table 3). Moreover, the concentrations of alanine aminotransferase (ALT), TG and CH all increased slightly after the sea voyage, but these differences were not significant.

## Discussion

In this study, volunteers worked on a large sailing ship for more than three months. The voyage took place in a maritime climate with high temperatures and humidity during the three-month voyage. In this type of environment, sailors suffer from dizziness, nausea and a series of physiological and psychological symptoms resulting in declined physical function.

Microbes co-exist in and on the human body and greatly impact human health and disease. To date, the metagenome has been extensively studied, and the relationship between the dysbiosis of the microbiome and worsened physical condition is becoming more evident. However, the relationship between the microbiota and a long sea voyage has not been fully explored. In this study, we surveyed the effects of a long sea voyage on the structure and metabolic condition of the buccal mucosa and the belly button commensal microbes by sequencing 16S rRNA and WGS.

Diversity is important in all ecosystems to promote stability and performance^17^. More diversity is considered to be more advantageous because of higher resiliency. The diversity of the microbiota may become a new biomarker or indicator of health^18^. Reduced microbial diversity in the gut has been reported to be associated with inflammatory bowel disease (IBD)^19^,^20^. Moreover, it is reported that individuals with a lower bacterial richness are characterised by more striking overall adiposity, insulin resistance and dyslipidaemia and a more noticeable inflammatory phenotype^21^. The human commensal microbiota is under direct influence of the external environment. The loss of biodiversity in the external environment correlates with atopic and chronic inflammatory diseases and may further affect the human commensal microbiota and its immune modulatory capacity^22^. Compared with a similar individual on land, the microbiota of an individual on a sailing ship has a much lower biodiversity. Although not significant when analysed using 16S rRNA sequencing (Supplementary Figure S4), our findings showed a much lower microbial diversity within an individual after a sea voyage using WGS. No significant change was observed in the microbial diversity according to 16S rRNA sequencing, most likely due to the PCR amplification of the 16S rRNA gene before sequencing that can lead to a failure in detecting low-abundance species. Further confirmation of the influence of a long sea voyage was provided by sequencing the microbiota of the belly button from the same individuals. The microbial diversity in the belly button samples was significantly reduced after the sea voyage. Thus, exposure to the sea voyage environment can reasonably be assumed to be one of the causes of reduced microbial diversity in sailors. Previous reports have suggested methods to increase the diversity of the commensal microbiota. Short-term probiotic administration was shown to enhance the microbial diversity in the saliva microbiome^23^. In another study, exercise and high protein intake were considered to be associated with higher microbial diversity^17^. Therefore, in preparing for a sea voyage, the sailing preparation, period and route should be carefully considered. Recommendations for long voyages include adding protein-rich food and food containing probiotics to the food inventory on the ship, docking as often as possible to allow sailors contact with an environment with higher biodiversity and thus enable the individual microbial diversity to be maintained at a relatively high level.

The oral microbiome plays an important role in human health. Approximately 1000 species are estimated to colonise the human mouth, making it the second most complex area of the human body^8^. The most abundant genera in the buccal mucosa are *Streptococcus*, *Haemophilus*, *Prevotella*, and *Veilonella*^8^. In our study, *Streptococcus* was the most abundant, whereas other genera were not as abundant before voyage, and which was increased after sea voyage. A large number (>100 species) of *Streptococci* colonise human and animal mucous membranes. However, numerous *Streptococci* act as opportunistic pathogens, causing infections only in the presence of a weak immunological response by the host^24^. Not limited in habitat, *Streptococci* in the oral cavity have a close relationship with the entire body. Group A *Streptococci* have long been associated with the development of autoimmune sequelae associated with rheumatic fever^25^. The primary manifestations of rheumatic fever involve the heart, joints, brain, and/or skin. Rheumatic carditis is the most serious of the five streptococcal sequelae and presents with a heart murmur as a result of valve deformation^26^. *S. sanguinis, S. mitis*, and *S.* *gordonii* have also been reported to be correlated with endocarditis^27^,^28^,^29^. *S.* *gordonii* is also involved in septic arthritis^30^. Moreover, in the after sea voyage samples, we discovered an increase in the number of species such as *S. cristatus, S. pneumoniae* and *S. anginosus*. Zhang *et al*. reported that *S. cristatus* attenuates the production of IL-8 stimulated by *Fusobacterum nucleatum* by blocking NF-kappa B nuclear translocation at the level of I kappa B-alpha degradation^31^,^32^. *S. pneumoniae* is a pathogen that is responsible for a variety of infections, including meningitis, otitis media, bacteraemia, and pneumonia. According to an estimation by the World Health Organisation (WHO), *S. pneumoniae* is the main cause of pneumonia, a disease that kills annually approximately 1.2 million children under 5 years old, which accounts for 18% of all deaths in this age group^33^. *S. anginosus* is part of the human bacteria flora, but it can cause infection under certain circumstances. The appearance of, and increase in, opportunistic pathogens indicate a weaker immune system, suggesting the necessity for creating a health management plan that includes physical exercise, sufficient rest, and a balanced diet to avoid the potential diseases caused by pathogens. Furthermore, it is reported that upper respiratory infections are among the highest morbidity diseases during sea voyages^1^, which is consistent with the observation of increased pathogens. However, we could not verify the direct correlation between increased pathogens and diseases during the voyage. Because opportunistic pathogens cause respiratory diseases and the air in the ship is recirculated, regularly examining the air circulation system of ships, including air conditioners, is important to avoid an epidemic outbreak during a sea voyage.

An unexpected result was that a large number of domain-level sequences remained unclassified (69.46%) in the after sea voyage sample. This finding was likely because these sequences were from ocean bacteria with genomes that are not in the database. Thus, this finding provides a new field that involves the exploration of the environment of the ocean and its influence on human health. With a deeper understanding of unclassified bacteria, we could better recognise the factors of a long sea voyage that influence the health of sailors.

As human's “second genome”, the genes of the metagenome encode a wide variety of proteins, which participate not only in the proliferation and metabolism of commensal microbiota but also in the metabolism and immune response of the human body. A previous metagenomic study of skin bacteria indicated that commensal *Staphylococcus* bacteria were enriched in mannitol utilisation, methicillin resistance, the alpha-acetolactate operon, triacylglycerol metabolism, acetoin metabolism, butanediol metabolism and arginine deiminase pathways, suggesting that these microbes are strongly adapted to the exploitation of compounds produced by the human skin^34^, including sugars, lipids and iron, and by petrobactin-mediated iron uptake systems. TCA-dependent ROS have been demonstrated to enhance the susceptibility of bacteria to antibiotics, and a propensity for clinical isolates to accumulate TCA cycle dysfunctions presumably as a way to tolerate antibiotics has been revealed^35^. In the buccal mucosa samples in our study, genes annotated with functions and projected to the KEGG pathways were greatly reduced after the sea voyage. The pathways with decreased expression included the TCA cycle and the three basic metabolic pathways, for carbohydrates, lipids and amino acids. In particular, genes involved in the urea cycle were reduced after the sea voyage. Continuously secreted from saliva and gingival crevicular fluid, urea is rapidly hydrolysed by bacteria in the oral cavity, resulting in ammonia production. The ammonia increases the pH of the oral environment, further changing the plaque biofilm and reducing the occurrence of saprodontia. Thus, the low frequency of genes involved in the urea cycle implies an increased incidence of saprodontia. Genes involved in nutrient metabolism detoxification were also reduced. Insufficient nutrient intake, especially of vitamins, is common during long sea voyages, which has induced diseases such as scorbutus for hundreds of years. Our discoveries revealed a decline in the metabolism or biosynthesis of ascorbate, carotenoid, and vitamin B groups such as folate, riboflavin, and thiamine, reflecting the insufficiency of certain nutrients in the human body. The data above indicate that a necessary step involves reserving ample nutrient supplements on sea voyage ships to prevent these diseases. Furthermore, xenobiotic biodegradation and metabolism correlates with the process of detoxification, in which microbes play important roles. Microbes degrade xenobiotics, which are usually caused by the release of industrial compounds. It is not surprising to find a decreased metabolic activity of xenobiotic biodegradation in the marine environment, where there is little industrial pollution. Moreover, investigation of the microbial metabolic activity in the belly button corroborated the results observed in the buccal mucosa. Finally, the vast majority of metabolites in our bodies are believed to not be of human origin, and thus a change in microbial metabolic activity directly influences human health^6^. However, we lack a clear understanding of whether microbial metabolites influence host biology. This area is therefore promising for future research. As a cofactor, folate is an essential nutritive component of the human diet, providing one carbon donor molecule for the biosynthesis of methionine, purine, and pyrimidine. Epidemiological studies have indicated that a folate deficiency is often associated with an increased risk of breast cancer^36^. Disrupted folate homeostasis may induce the hypomethylation of DNA, thereby promoting cancer in the proliferating cells of the colorectal mucosa^37^. Humans take in folate from their diet and from commensal bacteria mainly located in the colon. Some *Lactobacilli* and other lactic acid bacteria (LAB) contribute most to the synthesis of microbial folate in their human host^38^. The microbial metabolic analysis revealed that enzymes in the folate biosynthesis pathway were much less abundant in the after sample than in the before sample, implying that less microbial folate was synthesised. In accordance with this observation, our results suggest that *Lactobacillus lactis*, a major contributor of microbial folate, was reduced after the sea voyage (Figure 3c), suggesting that less microbial folate was produced for the host. Consistently, the medical examination of the seafaring sailors showed that the level of folate in the serum decreased after the ocean voyage. Therefore, evaluating the physical condition of an individual by examining the commensal bacteria is reasonable. Furthermore, bacterial folate plays a role in the host immune system. Kjer-Nielsen^39^ reported that microbial metabolites derived from folate and riboflavin are ligands for MR1, a molecule that binds and activates mucosa-associated invariant T (MAIT) cells. Thus, MAIT cells use these metabolites to detect microbial infection. Reduced microbial folate could potentially lead to a blind area of microbial infection detection and further influence the host immune response.

Another source of FA for humans is their diet. FA exists in fruits and vegetables and is especially rich in green-leaf vegetables, although these foods are difficult to store long-term. As a result, very few fruits and vegetables are consumed during a long sea voyage, leading to FA deficiency in sailors. Our data suggested that FA deficiency could be moderated if ships reached shore regularly to replenish fresh fruits and vegetables and if sailors took a vitamin supplement to provide FA. Thus, these measures are of great importance to ensure that the crew remains healthy. In contrast to FA, VB_12_ exists in meat and eggs, which are convenient for long-term storage, and thus the VB_12_ supply is adequate, which explains why no significant differences were observed between sailors and onshore workers. The analysis of HCY showed that HCY was inversely related to FA, a critical cofactor for HCY metabolism. High HCY levels in the blood leads to cardiovascular^40^ and other diseases, and FA intake is the perfect therapy^41^. Therefore, providing plenty of FA is a key step in health management for long sea voyages.

The results of the medical examination of a larger cohort who experienced the same voyage as the volunteers provided another hint regarding the physical condition of sailors. We discovered a decrease in the MCHC. In human adults, red blood cells (RBCs) differentiate from hematopoietic stem cells (HSCs) and mature in the niche of the bone marrow. VB_12_ and folate are critical materials for the maturation of RBCs. Combined with the decrease in the genes associated with the biosynthesis of folate in the metagenome and the relatively constant serum concentration of VB_12_ in sailors (Table 3), it is reasonable to deduce that the inactive biosynthesis of folate in the metagenome may have led to the reduced MCHC. Moreover, the decrease in GRN/LYM indicated a change in the immune system, and the slight increase in ALT indicated a change in liver function. Although the increases in TG and CH were not significant, the tendencies were consistent with a previous report that showed the TG and CH levels were higher in sailors than individuals on land^3^. The co-occurrence of increased lipid metabolites in the blood of sailors and the decrease in microbial lipid metabolism suggests a potential relationship between the host and commensal microbial metabolism. One possible reason of the elevated TG and CH is the relatively higher fat diet on the ship due to the lack of fresh fruits and vegetables. Elevated TG and CH indicated increase risk of obese. Accordingly, in our data it is found that an opportunistic pathogen, Enterobacteriaceae, which is reported to induce obesity in germ-free mice model^42^, increased after the long sea voyage. However, more data are needed to confirm the above speculation.

## Conclusions

In this study, we employed both 16S rRNA sequencing and WGS to clarify the impact of a long sea voyage on the buccal mucosa and belly button commensal microbiota. We investigated the oral commensal microbiota of sailors who experienced voyages of more than three months with highly intense work, altered diet and circadian biorhythms in an extremely humid and salty environment. Our data illustrated that the microbial diversity was reduced, several pathogens appeared, and the activities of microbial carbohydrate, lipid and amino acid metabolisms were lower. Microbial folate biosynthesis decreased sharply. Taxa analysis showed a large number of unclassified bacteria. Clinical analysis of the cohort showed reduced FA, MCHC and GRN/LYM and increased TG and CH, which are correlated with the changes in the commensal microbiota. Our discoveries indicate that the oral bacteria may reflect the health condition of the host. During a long sea voyage, the natural and working environment could weaken the human immune system and potentially cause disease. However, it is important to note that the analyses described above are a preliminary pilot project with a limited sample size and that more extensive studies are needed to confirm these observations. This article highlights, for the first time, the discovery of a correlation between the haemostasis of the oral mucosa bacteria and human health. Moreover, we analysed the influence of a long sea voyage on human health by examining the commensal microbiota using metagenomic approaches. Future work will focus on the exact factors in the sea voyage that cause the changes in the commensal microbiota on the basis of the metagenomic analysis of a larger sample size.

## Methods

### Voyage scope and volunteer information

All relevant aspects of the study were approved by the Institutional Review Board of the Cancer Hospital and Institute, Chinese Academy of Medical Sciences (CAMS) and Navy General Hospital (NGH), Beijing, China. Informed consent was obtained from each subject, and the study protocol was in accordance with ethical guidelines of the 1975 Declaration of Helsinki. A cohort (Cohort 1) undertook a voyage lasting a total of 105 days, covering a distance of 24,620 nautical miles. The first group in this cohort contained 57 individuals, including 48 males and 9 females, with a median age of 34 years (Cohort 1-Group 1) and received routine blood and biochemistry examinations including MCHC, RGN/LYM, ALT, TG and CH levels on the day before setting sail (before sea voyage) and on the day of docking (after sea voyage). The second group in cohort 1 (Cohort 1-Group 2), containing 24 individuals and having a median age of 25, was chosen for detecting serum concentrations of FA, HCY and VB_12_. In a third group, microbial samples were collected from 13 volunteers (Cohort 1-Group 3) from the cohort, with an average age of 29 (Supplementary Table S3). Microbial samples were collected at the same time as the above mentioned clinical examinations were performed.

The second cohort (Cohort 2) experienced a long sea-voyage and comprised 147 male individuals with an average age of 23.61 years. Cohort 2 was divided into 4 groups according to the duration of their voyage. Group A (30 individuals; sailing time, 120 days) and Group B (35 individuals; sailing time, 140 days) did not reach any port during their voyage. Group C (50 individuals; sailing time, 158 days) and Group D (20 individuals; sailing time 150 days) reached shore every 30 days to replenish the on-board supplies of fresh fruits and vegetables and took vitamin complex tablets during the sea voyage. The control group (12 individuals) was composed of onshore workers with an average age of 21.33 years.

### Microbiota collection

Microbiota samples were collected from the buccal mucosa and the belly button of volunteers before and after the sea voyage. The buccal mucosa and belly button samples were collected according to previous reports^43^,^44^. The swabs were drawn immediately after sampling, and the samples were frozen at −20°C without culturing and finally transferred to a −80°C freezer.

### DNA extraction

The stored samples were thawed on ice and a 1.5-ml bacteria suspension (a total of 2.5 ml) was vortexed and used for DNA extraction. The standard E.Z.N.A bacteria DNA isolation kit (Omega Bio-tek, Norcross, GA, USA) was used to extract DNA following the manufacturer's instructions. The genomic DNA quality and concentration were analysed by gel electrophoresis and the Nanodrop8000 (Thermo Electron Corp., Waltham, MA, USA), respectively. All the extracted DNA samples were stored at −20°C until further use.

### 16S rRNA sequencing and data analysis

16S rRNA sequencing was conducted on an Illumina MiSeq platform according to a protocol described previously^45^. PCR amplifications were conducted with the 515f/806r primer set, which amplifies the V4 region of the 16S rRNA gene. The reverse primer contains a 6-bp error-correcting barcode unique to each sample. DNA was amplified following the protocol described previously^46^. Pairs of reads from the original DNA fragments were merged using FLASH, and sequences were analysed using the QIIME software package and UPARSE pipeline^47^,^48^. Sequences were assigned to OTUs at 97% similarity; we selected a representative sequence for each OTU and used the RDP classifier to assign taxonomic data to each representative sequence.

### WGS and phylogenetic analysis

The Ion Torrent (Life Technologies, Carlsbad, CA, USA) and Illumina MiSeq sequencing systems (Illumina, San Diego, CA, USA) were used for sequencing, and the library preparation kits were purchased from New England Biolabs (New England Biolabs, Ipswich, MA, USA). Sequencing library construction and template preparation were performed according to the NEB library preparation protocols. We constructed a paired-end library with an insert size of ~300 bp for each sample. The Metagenomics RAST server (MG-RAST, release 3.3) was selected to analyse the data^49^. The uploaded data were subjected to the quality filter containing dereplication and removing host DNA by screening according to a previous report^50^,^51^. Determination of α-diversity and rarefaction was performed within MG-RAST by applying the “Best Hit Classification” option using the M5NR data base as a reference with the following settings: maximum *e*-value cutoff of 1 * 10^−5^, minimum identity of 80%, and minimum alignment length of 15 amino acids for proteins and 15 base pairs for RNA. The phylogenetic origin of the metagenomic sequences was projected against the NCBI taxonomic tree and determined by the lowest common ancestor (LCA) with the same cutoff mentioned above. The functional profiles were annotated according to KEGG Orthology (http://www.genome.jp/kegg/) with the same cutoff threshold mentioned above.

### Clinical data analysis

Routine blood examinations and biochemical examinations were performed according to regular laboratory test procedures.

### Folate, vitamin B12 and homocysteine detection

The detection kits for folate, vitamin B12 and homocysteine were all purchased from Roche (Basel, Switzerland) and used according to the manufacturer's protocol.

### Statistical analysis

The results are displayed as the means ± SD. Data analyses were performed using SPSS 19.0 software (SPSS Inc., Chicago, IL), and comparisons were based on independent sample t-tests.

Further additional figures and tables are described in the SI.

## Author Contributions

X.Z. designed research; Z.Z., C.L., W.Z., J.Q., D.Z. and M.X. performed research; Y.Q., H.A. and Z.M.Z. collected samples; W.Z. and Z.Z. analysed data; W.Z., Z.Z. and X.Z. wrote the paper. All authors reviewed the manuscript.

## Supplementary Material

Supplementary Information Zheng et al Supplementary Information

## Figures and Tables

**Table 1:** Comparison of high-abundant bacterial species in buccal mucosa samples before and after long sea-voyage operation

Domain	Phylum/Class/Order/Family	Genus	Species	Abundance* (%)	
				Before	After
Bacteria	Streptococcaceae	*Streptococcus*	-	65.93	54.29
Bacteria	Streptococcaceae	*Streptococcus*	*Streptococcus cristatus*	3.14	13.93
Bacteria	Streptococcaceae	*Streptococcus*	*Streptococcus pneumoniae*	1.62	6.98
Bacteria	Firmicutes	-	-	0.49	5.08
Bacteria	Streptococcaceae	*Streptococcus*	*Streptococcus mitis*	1.67	3.31
Bacteria	Lactobacillales	-	-	1.30	2.84
Bacteria	Streptococcaceae	*Streptococcus*	*Streptococcus anginosus*	0.00	1.65
Bacteria	Streptococcaceae	*Streptococcus*	uncultured *Streptococcus*	0.00	1.38
Bacteria	Streptococcaceae	*Streptococcus*	*Streptococcus* sp. C300	0.15	1.35
Bacteria	Bacilli	-	-	0.62	1.16
Bacteria	Pseudomonadaceae	*Pseudomonas*	*Pseudomonas aeruginosa*	4.09	0.00
Bacteria	Acholeplasmataceae	*Candidatus Phytoplasma*	*Aster yellows phytoplasma*	2.35	0.00
Bacteria	Pseudomonadaceae	*Pseudomonas*	-	1.75	0
Bacteria	Corynebacteriaceae	*Corynebacterium*	-	1.50	0.00
Bacteria	Moraxellaceae	*Acinetobacter*	-	1.20	0

*, The percentage of the certain taxa in the bacteria which were annotated at least at the phylum level. Top 10 abundant taxa in the before-sample or after-sample are shown.

**Table 2:** Effect of long sea-voyage operation on serum folic acid, vitamin B12 and homocysteine of sailors

Group	n	Folate Acid (μg/L)	Vitamin B_12_ (ng/L)	Homocysteine (μmol/L)
Control	12	6.80 ± 1.45	457.02 ± 151.26	12.96 ± 1.99
Group A	30	4.83 ± 1.86^a^	455.28 ± 167.30	17.64 ± 7.17^c^
Group B	35	5.30 ± 1.66^b^	431.72 ± 151.62	14.88 ± 6.47^c^
Group C	50	6.97 ± 2.47	402.67 ± 165.06	13.88 ± 5.82
Group D	20	7.42 ± 5.24	N/A	12.90 ± 5.19

Data from Cohort 2, including control group, onshore workers; Group A, sailors experiencing voyage without mooring at port; Group B, sailors experiencing voyage without mooring at port; Group C, sailors experiencing voyage with mooring at port; Group D, sailors experiencing voyage with mooring at port, n = 20. The *p* values are described as a, compare to the control, *p* = 0.0170; b, compare to the control, *p* = 0.0218; c, compare to the control, *p* < 0.001. Mann-Whitney test.

**Table 3:** The biochemical indexes of the cohort of the sailors

Biochemical index	Before	After	*p* value	Significant
MCHC (g/L)	332.57 ± 10.29	314.81 ± 11.67	<0.001	Yes
GRN/LYM	1.71 ± 0.63	1.35 ± 0.46	<0.001	Yes
ALT (u/L)	29.58 ± 21.84	34.37 ± 27.96	0.321	No
TG (mmol/L)	1.38 ± 0.9	1.57 ± 0.96	0.318	No
CH (mmol/L)	4.63 ± 0.80	4.71 ± 0.91	0.572	No

MCHC, mean corpuscular haemoglobin concentration; GRN/LYM, ratio of neutrophils to lymphocytes; ALT, alanine aminotransferase; TG, triglyceride and CH, cholesterol. Data from Cohort 1-Group 1 including 57 individuals (48 males and 9 females, with a median age of 34 years), which received a routine blood and biochemistry examinations including MCHC, RGN/LYM, ALT, TG and CH levels on the day before setting sail (before) and the day of docking (after).
