# Supplementary material for: Metagenomic sequencing reveals altered metabolic pathways in the oral microbiota of sailors during a long sea voyage
Source: Sci Rep. 2015 Mar 16;5:9131. doi: 10.1038/srep09131 (PMC4360635; doi:10.1038/srep09131)
Supplement: Supplementary Information — Zheng et al Supplementary Information [file srep09131-s1.pdf]

Supplementary Information

Title: Metagenomic sequencing reveals altered metabolic pathways in the oral microbiota of crewmembers during a long sea voyage

Authors: Weiwei Zheng[1#], Ze Zhang[2#], Cuihua Liu[3#], Yuanyuan Qiao[1#], Dianrong Zhou[2], Jia Qu[1], Huaijie An[1], Ming Xiong[1], Zhiming Zhu[1], Xiaohang Zhao[1,2,4*]

Affiliations: [1]Center of Basic Medical Sciences, Navy General Hospital, Beijing, China; [2]Third School of Clinical Medicine, Southern Medical University, Guangzhou, China; [3]School of Life Science, Tsinghua University, Beijing, China; [4]State Key Laboratory of Molecular Oncology, Cancer Institute and Hospital, Chinese Academy of Medical Sciences and Peking Union Medical College, Beijing, China

Additional Footnotes: [#]these authors contributed equally to this work.

Corresponding author: Dr. Xiaohang Zhao, State Key Laboratory of Molecular Oncology, Cancer Institute and Hospital, Chinese Academy of Medical Sciences and Peking Union Medical College, Beijing, China; E-mail: zhaoxh@cicams.ac.cn.

Supplementary Figure Legends

Supplementary Figure S1. Pie chart of the taxonomic annotation levels of sequencing.

The taxonomic annotation level of the samples from 16S rRNA sequencing (a) and whole-genome shotgun sequencing (WGS, b) is shown. For 16S sequencing, the data was analysed by Qiime software package and UPRASE pipeline. For WGS, the data was analysed by MG-RAST server through Lowest Common Ancestor method using a maximum e-value of 1e-5, minimum identity of 80%, and minimum alignment length of 15 in amino acid in protein and bp in RNA databases.

Supplementary Figure S2. 16S sequencing analysis of variation of the belly button microbiome before and after a long sea voyage.

Pairwise samples were collected at the time of setting sail and docking, n=12. The data was analysed by Qiime software package and UPARSE pipeline. (a and b) Pie chart of the relative abundance of the bacterial taxonomic hits at the phylum level in belly button samples before (a) and after the sea-voyage (b). The data are shown as the means. (c) Bar chart of the relative abundance of bacterial taxonomic hits at the phylum level in the belly button before and after the voyage (For Firmicutes, p=0.0005; for Proteobacteria, p=0.0034). (d) Analysis of the changes in most abundant genera (For Staphylococcus, p=0.0093; for Corynebacteria, p=0.001). (c and d) The data are shown as means ± SD, Wilcoxon matched pairs test.

Supplementary Figure S3. Variation of the taxonomic hit distribution after a long sea-voyage analysed by WGS (belly button samples).

(a and b) Pie chart of the relative abundance of the MG-RAST taxonomic hits at the phylum level in buccal mucosa samples before (a) and after (b) a long sea-voyage. (c) Heatmap depiction of the relative abundance of bacterial species from buccal mucosa samples at the species level. The colours reflect the relative abundance from low (blue)

to high (red). The data was analysed through Lowest Common Ancestor method with a maximum e-value of 1e-5, minimum identity of 80%, and minimum alignment length of 15 bp in RNA databases.

Supplementary Figure S4. Alpha Diversity of 16S sequencing samples.

The alpha diversity of buccal mucosa or belly button samples are shown. Pairwise samples were collected from buccal mucosa or belly button at the time of setting sail (before, blue) and docking (after, red). Sequences were analyzed with the QIIME software package and UPARSE pipeline, in addition to custom Perl scripts to analyze alpha diversity. The alpha diversity is computed as Shannon diversity (species).

Supplementary Table S1. Statistic of 16S rRNA and whole genome sequence data.

Sequence Type	Body Site	Total Sequence	Qualified Sequence	Average Length(bp)	Average GC%
16S rRNA	Buccal Mucosa	55740	54219	253	51.80
	Belly Button	53617	52113	253	52.37
WGS	Buccal Mucosa	3804224	794145	134	47.5
	Belly Button	3840082	2151679	134	47

Supplementary Table S2. Information of detected enzymes on the folate biosynthesis pathway.

Enzyme Entry	Name	Hits-before	Hits-after	Class
EC 4.2.3.12	6-pyruvoyltetrahydropterin synthase	61	12	Lyases
EC 3.1.3.1	alkaline phosphatase	14	3	Hydrolases; Acting on ester bonds; Phosphoric-monoester hydrolases
EC 4.1.2.25	Dihydroneopterin aldolase	4	0	Lyases
EC 1.5.1.34	6,7-dihydropteridine reductase	1	0	Oxidoreductases
EC 2.7.6.3	2-amino-4-hydroxy-6-hydroxymethyl-dihydropteridine diphosphokinase	5	1	Transferases
EC 4.1.3.38	aminodeoxychorismate lyase	2	1	Lyases
EC 2.6.1.85	aminodeoxychorismate synthase	14	1	Transferases
EC 2.5.1.15	dihydropteroate synthase	74	1	Transferases
EC 6.3.2.17	tetrahydrofolate synthase	1	0	Ligases
EC 1.5.1.3	dihydrofolate reductase	45	1	Oxidoreductases

Supplementary Table S3. Information of volunteers for metagenomic sequencing.

No.	Nationality	Age	Height (cm)	Weight (Kg)	Smoke	Alcohol	16S rRNA	Deep sequence
P1	Han	29	172	79	Yes	Occasional	Yes	——
P2	Han	29	170	62	Yes	Yes	Yes	——
P3	Han	28	172	60	Occasional	Yes	Yes	——
P4	Han	21	173	65	Yes	Yes	Yes	——
P5	Han	24	170	76	No	No	Yes	——
P6	Han	25	179	82	Yes	Yes	Yes	——
P7	Han	37	176	82	Yes	Occasional	Yes	——
P8	Han	27	175	75	Yes	Yes	Yes	——
P9	Han	41	174	78	Yes	Occasional	Yes	——
P10	Han	23	175	75	Occasional	Occasional	Yes	——
P11	Han	23	175	60	Yes	Occasional	Yes	——
P12	Han	32	176	82	Yes	Occosional	Yes	——
P13	Han	38	164	54	No	No	——	Yes

Data from Cohort 1-Group 3 including 13 individuals (12 for 16S rRNA sequencing analysis and 1 for WGS).

Supplementary Figures

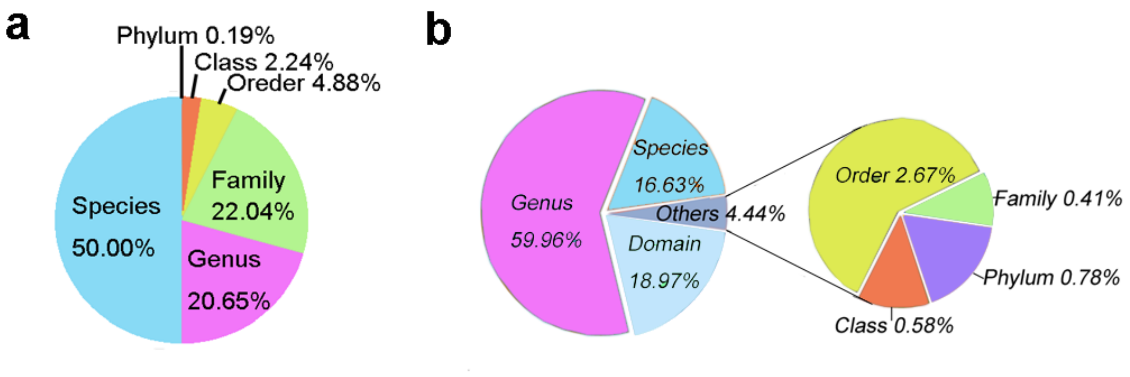

Supplementary Figure S1 Pie chart of taxonomic annotation level of sequencing.

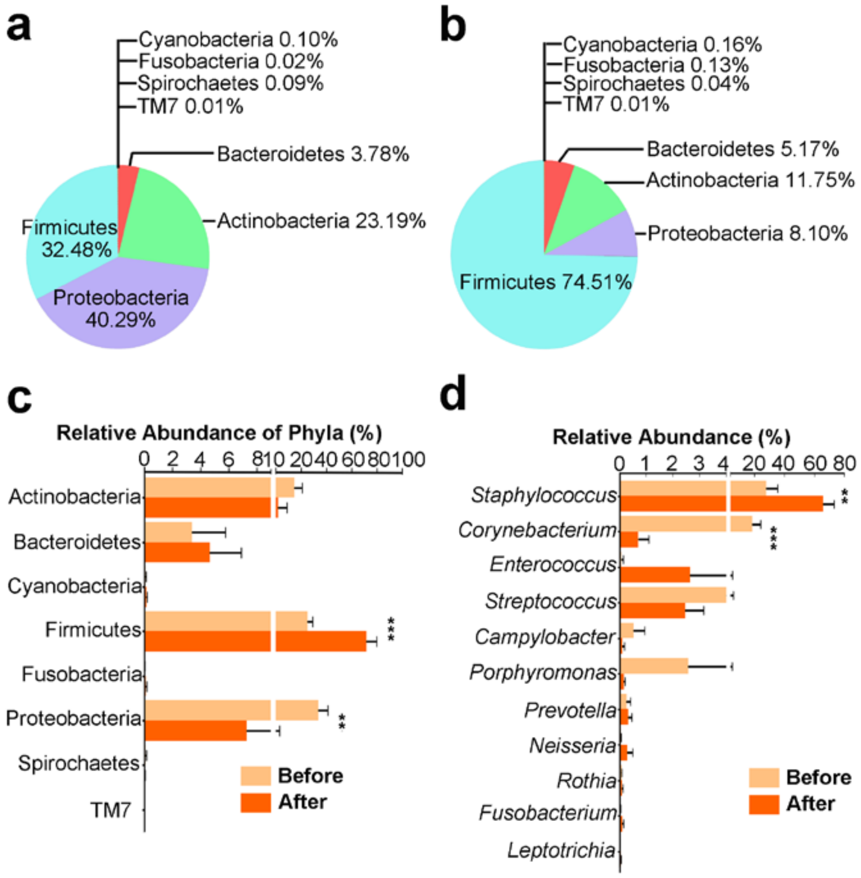

Suppleme mtary Figure S2 16S sequencing analysis of variation of belly button microbiome before and after long sea voyage.

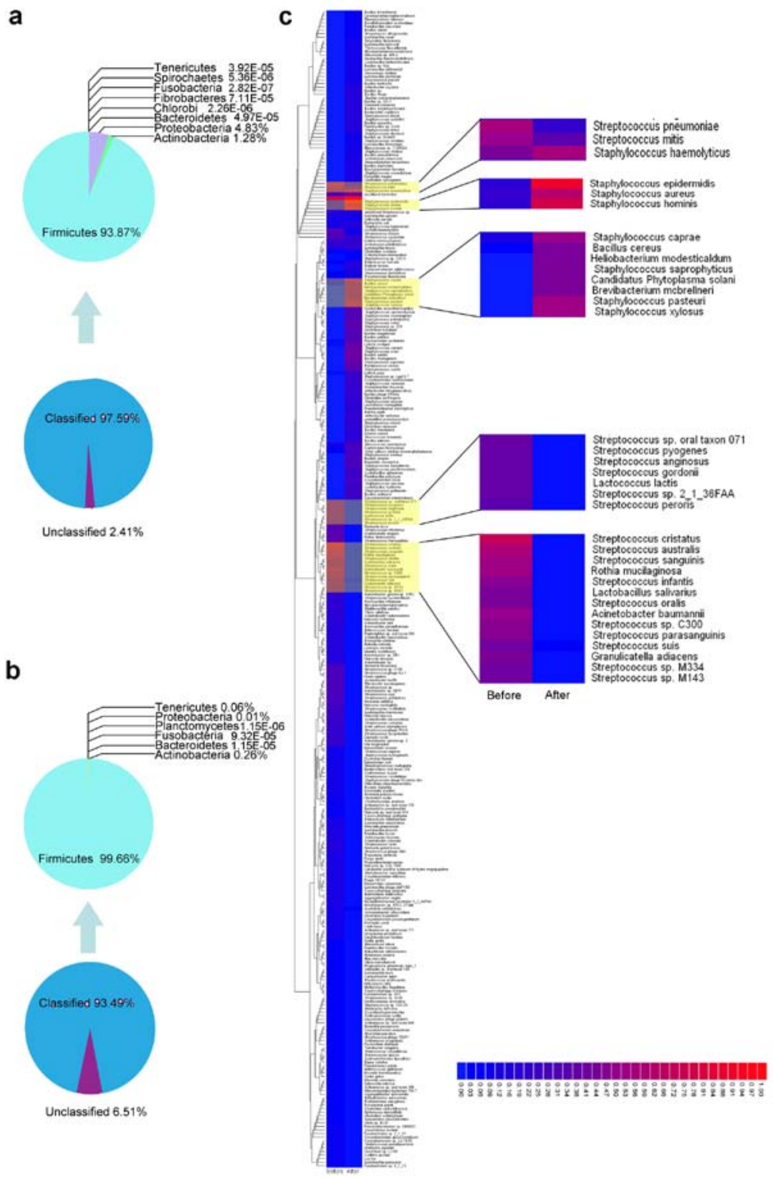

Supplementary Figure S3 Variation of taxonomic hits distribution after long sea-voyage operation in WGS (Belly Button).

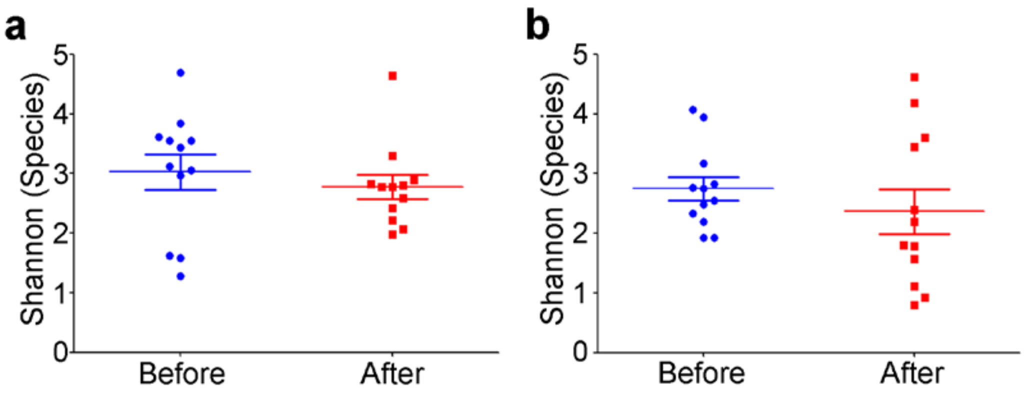

Supplementary Figure S4 Alpha Diversity of 16S sequencing samples.
